# Population Structure, Abundance and Movement of Whale Sharks in the Arabian Gulf and the Gulf of Oman

**DOI:** 10.1371/journal.pone.0158593

**Published:** 2016-06-30

**Authors:** David P. Robinson, Mohammed Y. Jaidah, Steffen Bach, Katie Lee, Rima W. Jabado, Christoph A. Rohner, Abi March, Simone Caprodossi, Aaron C. Henderson, James M. Mair, Rupert Ormond, Simon J. Pierce

**Affiliations:** 1 Heriot-Watt University, Edinburgh, United Kingdom; 2 Qatar Ministry of Environment, Doha, Qatar; 3 Maersk Oil Research and Technology Centre, Doha, Qatar; 4 Environment Department, University of York, York, United Kingdom; 5 Gulf Elasmo Project, Dubai, United Arab Emirates; 6 Marine Megafauna Foundation, Truckee, CA, United States of America; 7 Sharkwatch Arabia, Dubai, UAE; 8 School of Field Studies, Turks & Caicos Islands, South Caicos; 9 Marine Conservation International, Edinburgh, United Kingdom; Biodiversity Research Center, Academia Sinica, TAIWAN

## Abstract

Data on the occurrence of whale sharks, *Rhincodon typus*, in the Arabian Gulf and Gulf of Oman were collected by dedicated boat surveys and via a public-sightings scheme during the period from 2011 to 2014. A total of 422 individual whale sharks were photo-identified from the Arabian Gulf and the northern Gulf of Oman during that period. The majority of sharks (81%, n = 341) were encountered at the Al Shaheen area of Qatar, 90 km off the coast, with the Musandam region of Oman a secondary area of interest. At Al Shaheen, there were significantly more male sharks (n = 171) than females (n = 78; X2 = 17.52, P < 0.05). Mean estimated total length (TL) for sharks was 6.90 m ± 1.24 (median = 7 m; n = 296). Males (7.25 m ± 1.34; median = 8 m, n = 171) were larger than females (6.44 m ±1.09; median = 7 m, n = 78; Mann-Whitney U test, p < 0.01). Of the male sharks assessed for maturity 63% were mature (n = 81), with 50% attaining maturity by 7.29 m and 100% by 9.00 m. Two female sharks of >9 m individuals were visually assessed as pregnant. Connectivity among sharks sighted in Qatari, Omani and UAE waters was confirmed by individual spot pattern matches. A total of 13 identified sharks were re-sighted at locations other than that at which they were first sighted, including movements into and out of the Arabian Gulf through the Strait of Hormuz. Maximum likelihood techniques were used to model an estimated combined population for the Arabian Gulf and Gulf of Oman of 2837 sharks ± 1243.91 S.E. (95% C.I. 1720–6295). The Al Shaheen aggregation is thus the first site described as being dominated by mature males while the free-swimming pregnant females are the first reported from the Indian Ocean.

## Introduction

The whale shark, *Rhincodon typus* (Smith, 1828), is the world’s largest fish. This species is distributed throughout tropical and warm temperate seas [1]. The whale shark is one of three filter-feeding shark species, and preys on a variety of nektonic and planktonic organisms [1,2]. Although significant gaps in our knowledge of its biology still exist [3], the whale shark has been classified as Vulnerable on the IUCN Red List of Threatened Species [4] due to anthropogenic pressures, particularly directed fisheries in south-east and south Asia [5–10]. A challenge in conservation assessment to date has been a lack of knowledge on the population ecology of mature whale sharks.

Whale sharks form feeding aggregations in a number of regions around the world, including Western Australia [11], Belize [12], northern Mexico [13], the Philippines [14], Djibouti [15], Mozambique [16], Tanzania [17], the Maldives [18,19], the Seychelles [20,21], Red Sea [22] and Qatar [23]. All of these aggregation sites, excepting the Red Sea where the sex ratio is 1: 1 [22], are frequented primarily by juvenile males [15,19,24–27]. In most areas, the mean total length (TL) of sharks is between 6 and 8 m [27], the exceptions being Djibouti and the Red Sea where smaller mean sizes of 3.7 m and 4.0 m respectively have been documented [15,22]. In male whale sharks, visual assessment of claspers can be used to determine maturity [17,28]. Size at maturity for males varies from approximately 7 m in the Caribbean [29] to 8 m in Western Australia [28,29], with sizes estimated visually, to 9.16 m in Mozambique [27] estimated by laser photogrammetry [30]. Maturity in free-swimming female whale sharks cannot be assessed unless pregnancy is visibly indicated by a distended and swollen abdomen [31,32]. The few places in the world where pregnant female whale sharks have been documented include sites off the Pacific and northwestern Caribbean Sea coasts of Mexico [13,33], Taiwan [34], and around the northern Galapagos Islands off Ecuador [32,35]. Based on those limited data, size at maturity for females is approximately 9 m [36].

The use of photographic identification in elasmobranchs is a reliable and non-intrusive method of identifying individual animals and obtaining information about populations [37]. Taylor [38] took images of whale sharks at Ningaloo Reef, Australia in an attempt to use scars as a means of identification and found that the colour patterns on the sides of the sharks were unique and stable over time. It was concluded that these patterns could be used to confirm the identification of individuals. Norman [39] found that the area behind the fifth gill slit and above the pectoral fin was well-suited to identify individual whale sharks; two sharks were identified at Ningaloo over a 12-year period confirming stability for at least this period. Sightings and re-sightings of individuals within a population can be used in maximum likelihood models to estimate population size and residency [40–42]. The Lagged Identification Rate (LIR) metric, defined by Whitehead [43] as the probability of re-identifying an individual that was identified some lag time earlier, is a useful modelling approach for opportunistic sighting data and best-fit LIR models have now been widely applied to estimate population parameters [36,37,40,42,44].

Before 2010 there were few regional records of whale sharks in the literature from the Arabian Gulf and Gulf of Oman [45–51], with the majority reported in the local press. Here we show that the Al Shaheen area is in fact a globally-significant whale shark hotspot, and the first to be dominated by mature male whale sharks. Connectivity within the Arabian Gulf and Gulf of Oman is established, allowing us to provide the first regional population estimate for this globally threatened species.

## Materials and Methods

### Study area

**Fig 1:**
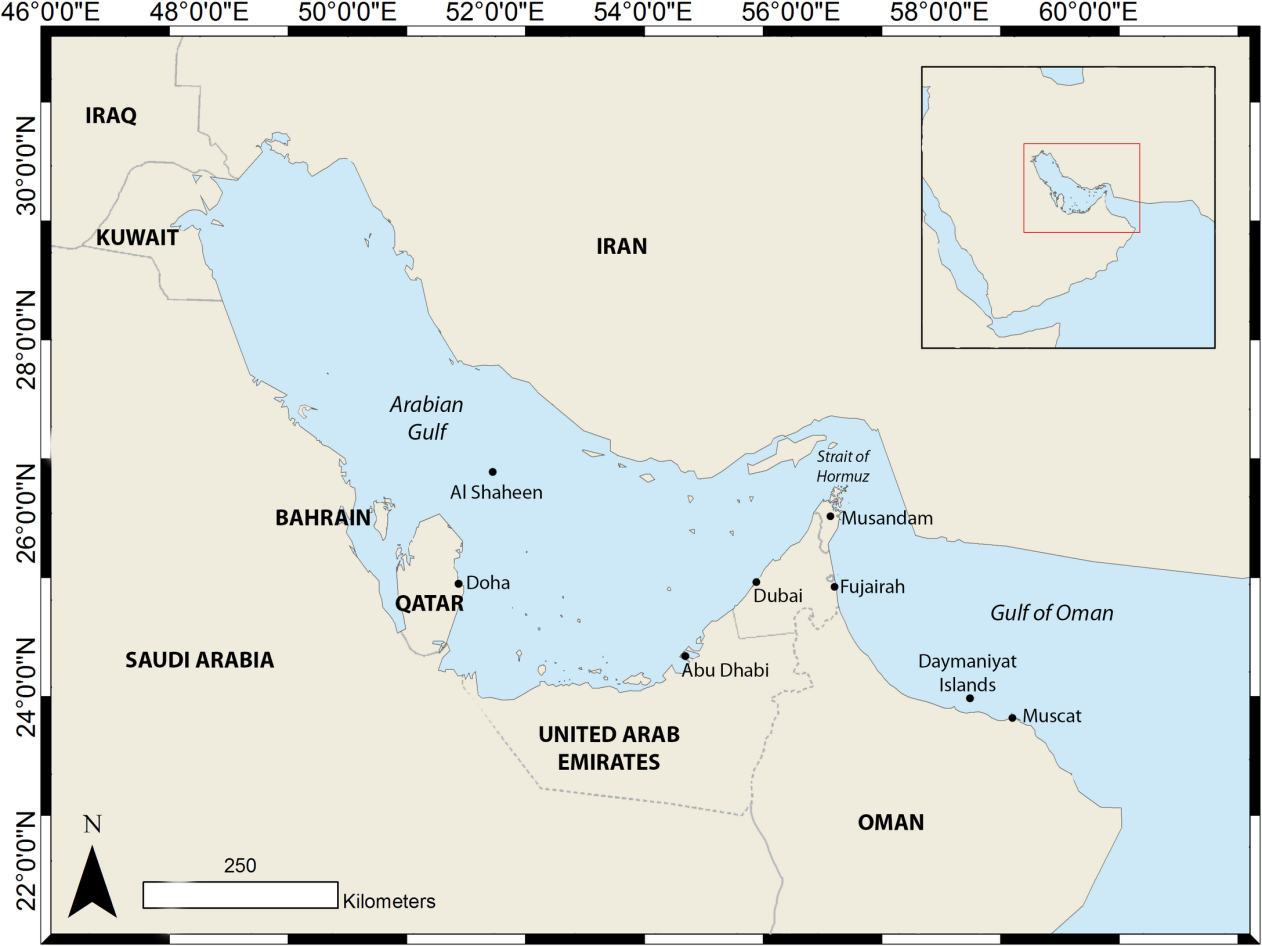
The locations of all study sites and other points of interest for whale sharks in the region, with the Arabian Peninsula (inset).

This study incorporates the Arabian Gulf, the Strait of Hormuz and North-West Gulf of Oman (Fig 1). This area is one of the most economically-important waterways in the world, with heavy ship traffic related to oil and gas transportation [52]. The Gulf of Oman is up to 2000 m deep while the Arabian Gulf is shallow throughout, with a maximum depth of just over 90 m [53]. Marine environmental conditions in the Arabian Gulf are among the most extreme on the planet [54], exposed to sea surface temperatures regularly exceeding 35°C, and reaching 40°C in some areas in mid-summer, and dropping to below 10°C during winter. The lack of precipitation and high evaporation rate results in salinity ranging from 28–60 ppt. The Gulf of Oman has more stable and moderate conditions, as the southwest monsoon causes cool-water upwellings. Surface temperature rarely exceeds 30°C and salinity is normal at around 35 ppt with little variation over the year [54].

### Data collection

Data were collected through public submissions of photographs and dedicated field studies. Seafaring individuals, such as fishers, oil platform workers, leisure divers and tour boat operators, were encouraged to submit any information on sightings of whale sharks in this region to the online forum Sharkwatch Arabia (www.sharkwatcharabia.com). Participants were requested to provide as much information as available about each encounter, including date and time, location, size, sex and the presence of associated fauna. All encounters were subsequently submitted to the Wildbook for Whale Shark database (www.whaleshark.org) to investigate broader-scale connectivity.

### Platform-based observations in Al Shaheen

Platform stationed staff working for Maersk Oil provided opportunistic observations of whale sharks in Al Shaheen. These sightings, often supported by video and photographs, were logged on a daily basis for four years from 2011 to 2014. The platforms are elevated, with 360° views of the surrounding waters. All workers were briefed to report sightings and to record the time of the sighting, along with the estimated number of sharks, to their designated sightings collator. One person stationed on each of the eight platforms was asked to collate the sightings on a daily basis from their platform workers and log every time a shark or group of sharks was observed. Only the maximum number of sharks observed per day in one group from one platform was used in the analysis to eliminate repeat observations and only sharks reported during daylight hours were used in the final analyses.

### Dedicated surveys in Al Shaheen

Permissions for fieldwork and data collection on whale sharks in the Al Shaheen region of Qatar were given by the Qatar Ministry of Environment. The whale shark has been classified as Vulnerable on the IUCN Red List of Threatened Species [4] but is not protected in Qatari waters where the fieldwork was carried out. Data collection was non-invasive using only photography.

Forty-one at-sea surveys took place during May to September: 7 in 2011, 16 in 2012 and 9 in each of 2013 and 2014. For safety reasons, no surveys could be conducted when wind speed exceeded 12 knots. Surveys were carried out from a 10 m vessel, powered by twin 250hp engines, which took approximately 2 hours to reach the study area, with the start time ranging from 5 to 9 am. Time in the field ranged from four to six hours. During each survey, a set route was followed among the eight fixed gas platforms. Whale sharks were detected from sightings of the first dorsal and or caudal fin breaking the surface of the water.

Upon sighting either an individual or aggregation of sharks, a GPS location and time was recorded and shark numbers were visually estimated from the boat. A team of 2–4 researchers then entered the water, using snorkelling gear and equipped with digital cameras. Researchers took photographs of the flank area on each shark behind the fifth gill slit and above the pectoral fin on the left side of the shark for the purposes of individual identification (IDs) [55]. The sex of each shark was determined by the presence (males) or absence (females) of claspers. Maturity status of male whale sharks was assigned based on the visual inspection of the length and thickness of claspers using the criteria described in Rohner et al. [27]. Pregnancy in female sharks was assessed using both estimated TL and the presence of a distinctive swollen abdomen as described in Acuña-Marrero et al. [32].

Images collected during fieldwork in Al Shaheen were processed for visual matching using I^3^S software [56] and also submitted directly to the online database Wildbook for Whale Sharks to look for matches from other observers. The length at which 50% of males reach maturity (TL50) was calculated using a generalised linear model (GLM) with a binary logit function.

### Estimation of total length

Where possible, the size of each animal was estimated, usually by comparison with the boat or another snorkeler. A subset of sharks was measured using laser photogrammetry. Green lasers (Sea Turtle Scuba Inc.) were placed 50 cm apart in a custom made steel frame and aligned in parallel. A camera in an underwater housing was placed between the two lasers in the middle of the frame. Lasers were calibrated each day before use by measuring the distance between the projection of the lasers against a marker at varying distances up to 10 m. Images were taken perpendicular to the sharks and only clear images which displayed the animal in a stretched position were used. Methods developed by Rohner et al. [27,30] were used to estimate total length from a measured body proportion. To test the accuracy of researcher length estimates, laser photogrammetry estimates were compared to the researchers’ visual estimates. Laser photogrammetry was performed throughout the 2012 season and 13 sharks had a suitable image for analysis as well as an independent visual size estimate.

### Spatial analyses

All whale shark encounter locations reported through the project were input to ArcGIS 10.2.1. The “kernel density tool” was used to calculate occurrence magnitude per km^2^. To determine areas of overall and core habitat usage, 50% and 95% Volume Contours (PVC) were produced. Both kernel density and PVC were produced following the methodology outlined in MacLeod [57].

### Regional population estimates

The residence time for individuals within the study area was investigated by using the “movement” module of SOCPROG 2.4 [58] to calculate the Lagged Identification Rate (LIR), the probability that an animal identified from an area will be re-sighted again after a variable lag time [40]. All whale sharks that had been individually identified from within or outside the Gulf from both dedicated fieldwork and submitted encounters were used for this analysis. All individuals were linked to a location from January 2010 to December 2014. The lowest value from quasi-Akaike information criterion (QAIC) values were used to select the best fitting residence model, accounting for over-dispersion of data [43].

The LIR analysis was extended to split the study area into two sub-areas: inside and outside the Arabian Gulf. The LIR then represented the probability that an individual identified within one area will be re-sighted in the other area after a specified time lag [40]. A fully-mixed model was fitted to the data as photo-ID results showed that some movement between areas did occur (see Results). The model also accounted for movements to a third, hypothetical area (i.e. outside the Gulf region). Data were bootstrapped for 100 repetitions to calculate confidence intervals (C.I.) and standard errors (S.E.)

## Results

### Sightings, photo-identification and seasonality

Three hundred and forty-one individual whale sharks were identified from 980 useable identification photographs taken during fieldwork in Al Shaheen from 2011 to 2014. Both sex and size were recorded for 249 individuals. A total of 4350 whale shark sightings were reported from Al Shaheen between 2011 and 2014 while only one other whale shark was reported outside of this area, from a man-made waterway in Doha in 2012.

**Fig 2:**
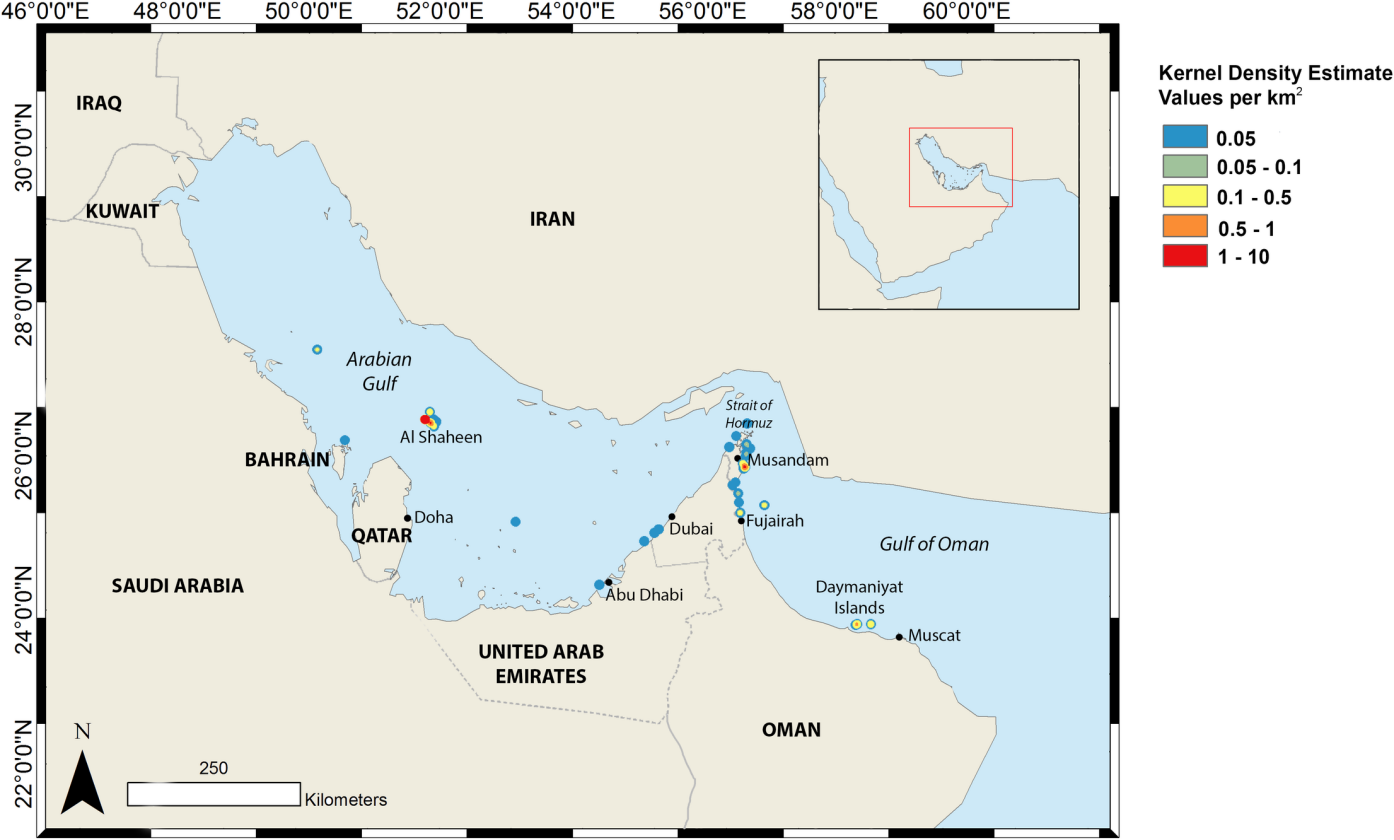
Kernel density analysis showing the overall regional occurrence of whale sharks based on submitted sightings.

Excluding Al Shaheen, 81 individual whale sharks were identified from the Arabian Gulf and Gulf of Oman from records starting in 2004, including the Musandam (n = 46) and Daymaniyat Islands (n = 27) in Oman, and Fujairah (n = 8) in UAE (Fig 2).

**Fig 3:**
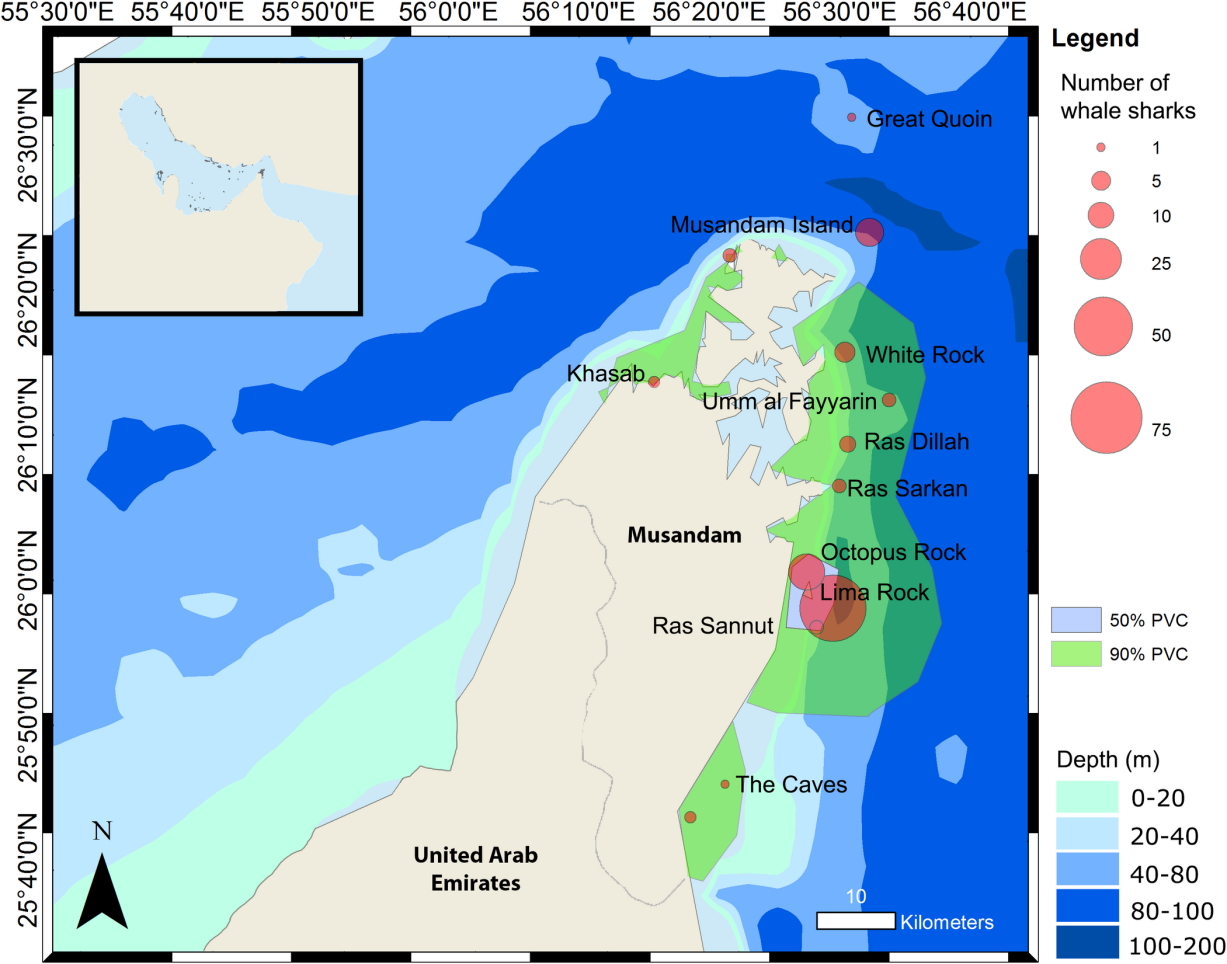
The frequency of whale shark encounters at different sites around the Musandam Peninsula, together with the 50% and 95% Percentage Volume Contours (PVC).

Ninety-five whale shark encounters were reported from the Musandam (Oman). Sightings were most frequent at Lima Rock (Fig 3) with 64 encounters, followed by Octopus Rock with 19 encounters. Fifty whale shark encounters were reported from the Daymaniyat Islands, with 19 encounters made at the dive sites ‘Junn’ and ‘Aquarium’ and nine sharks from ‘Sira’.

Forty-three whale shark encounters were reported from UAE waters. The majority of sharks were seen off Fujairah on the East coast, with 10 sharks encountered at the dive site Martini Rock followed by six sharks encountered at Dibba Rock. A feeding aggregation of approximately 10 sharks was reported by fishermen from 35 km off Fujairah in 2012. Twelve sharks were reported from inside the Arabian Gulf coast of the UAE; all but three of these sharks were reported from marinas and ports. Two sharks were encountered by members of the public in 2013 off Jumeirah Beach and one shark was reported from the Salman Oil Field about 100 km offshore of mainland UAE. Body size of sharks ranged between an estimated 4 and 6 m TL.

**Fig 4:**
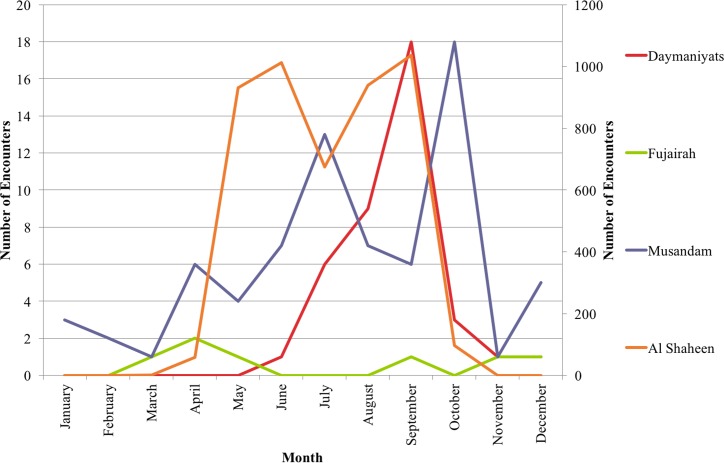
Regional whale shark encounters reported to the Sharkwatch Arabia project (Al Shaheen is shown on the right hand Y axis).

Most sharks, at all sites, were encountered in the summer months from April to October, although sharks were encountered year-round outside of Al Shaheen. Sharks seen from November to March were usually single individuals, while aggregations were common from April to October. This pattern was consistent with observations from oil platform workers in Al Shaheen (Fig 4).

### Population ecology in Al Shaheen

Sixty individual sharks were identified during 2011, 178 during 2012, 119 during 2013 and 128 during the 2014 season (Table 1). The percentage of re-sighted sharks varied between 13% and 59% with the mean inter-annual re-sight rate being 41%. Re-sights of sharks identified in previous years were seen in all subsequent field seasons from 2012 to 2014.

**Table 1:** Inter-annual re-sight rates (%) for individual sharks first identified in Al Shaheen.

Year	Identified sharks	*2011 re-sights (%)*	*2012 re-sights (%)*	*2013 re-sights (%)*	*Total re-sight (%)*
2011	60				
2012	178	*13*			***13***
2013	119	*21*	*38*		***59***
2014	128	*15*	*27*	*8*	***50***
*Mean re-site rate (%)*					***41***

**Fig 5:**
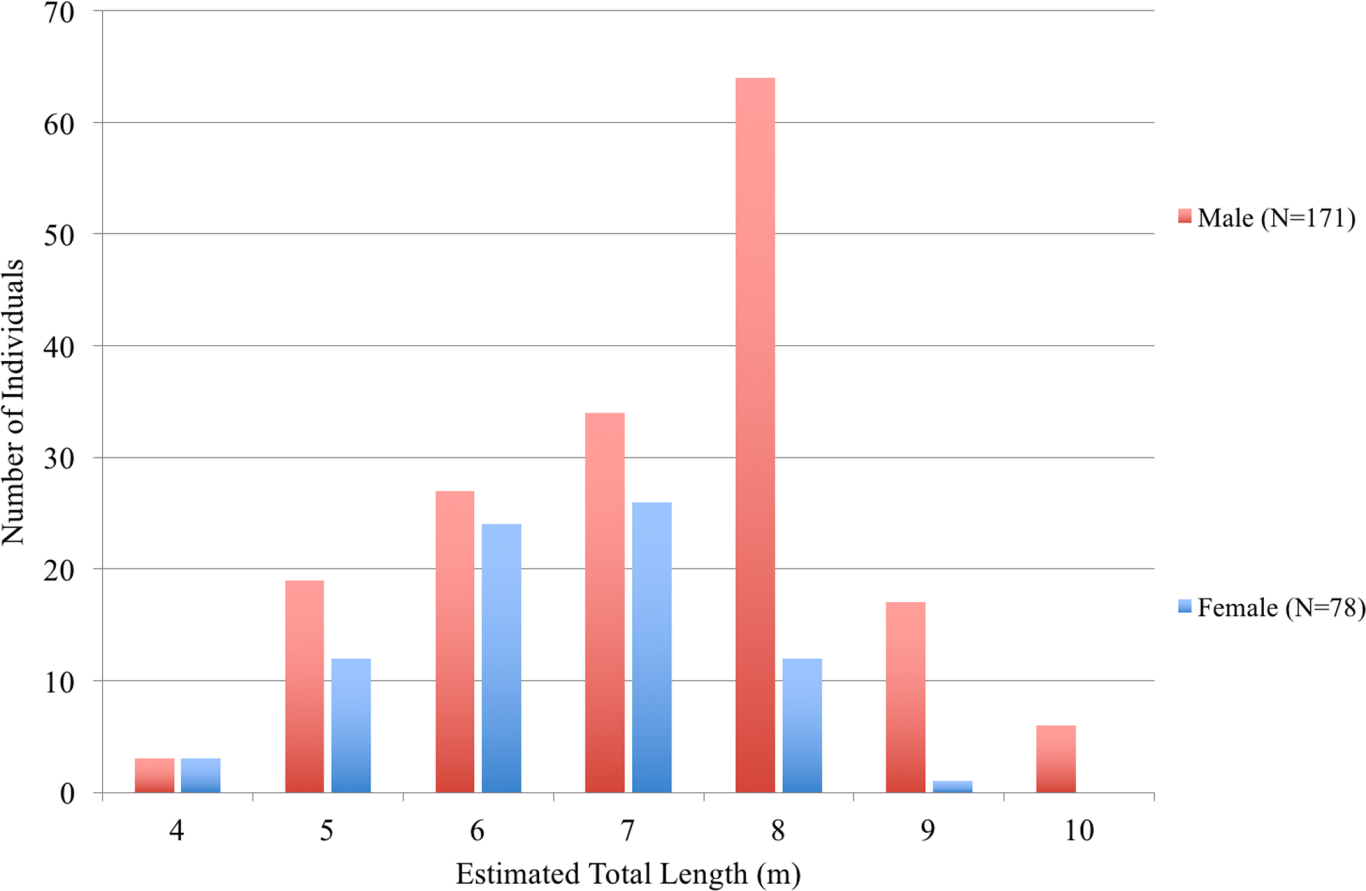
Visual size estimates of whale sharks from Al Shaheen.

There were significantly more male sharks (n = 171) than females (n = 78; X_2_ = 17.52, P < 0.05). Whale sharks at Al Shaheen ranged from 4 to 10 m TL (Fig 5), with an overall mean of 6.9 m ± 1.24 TL (median = 7 m, n = 296). Mean TL for males (7.25 m ± 1.34; median = 8 m, n = 171) was significantly larger than TL for female sharks (6.44 m ± 1.09; median = 7 m, n = 78; Mann-Whitney *U* test, p < 0.01). Mean shark TL from laser photogrammetry was 6.80 ± 1.10 m (median 6.74 m, n = 13; range 5.2–8.2 m). The corresponding mean TL for visual estimates of the same sharks was 7.00 ± 1.15 m (median = 7 m, n = 13; range 5–9 m). There was no significant difference in TL estimates derived from the two different techniques, with only one individual differing by >1.00 m.

In sharks where both sex and size were recorded, there was a notable male bias (69%, n = 249). Of the males assessed for maturity, 63% (n = 81) were mature. Males reached maturity between 7 and 9 m TL, with TL_50_ at 7.29 m (Chi^2^ test: d.f. = 79, Res. Dev = 41.996, p < 0.0001). All sharks were mature at or above 9 m TL. Two 9 m TL females had conspicuously distended and swollen abdomens, suggesting they were pregnant.

### Regional connectivity and population size

**Fig 6:**
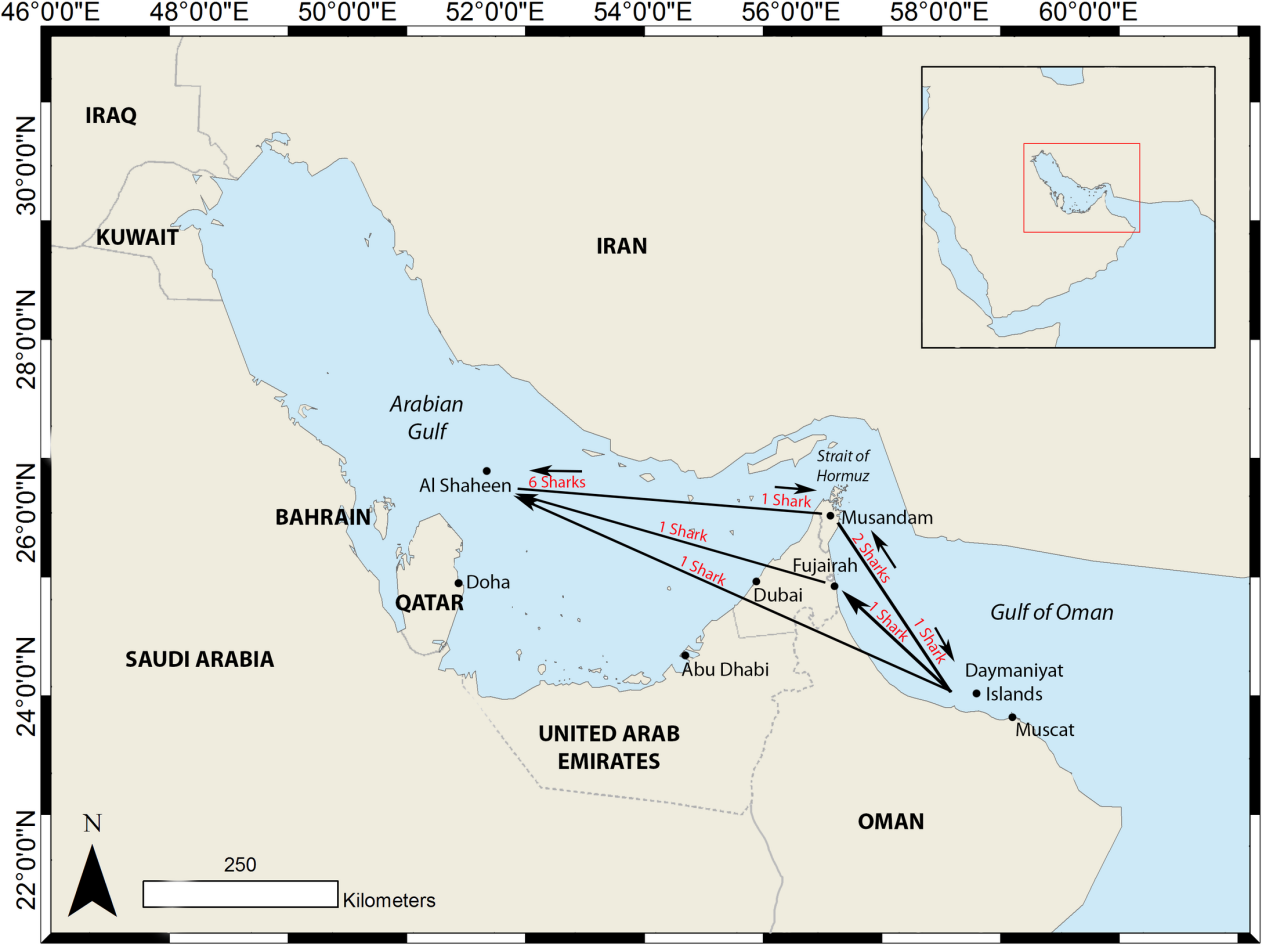
Movements of 13 whale sharks identified from their spot pattern and re-sighted at different locations from those at which they were first recorded.

Thirteen sharks were re-sighted at different locations from those at which they were first recorded (Fig 6). The majority of movements were between Musandam and Al Shaheen. The longest duration between a re-sighting was four years, for a shark seen in Fujairah in 2010 and re-sighted at Al Shaheen in 2014. The longest distance travelled was for a shark first identified in the Daymaniyat Islands (in 2012) and re-sighted in Al Shaheen (2014), an estimated straight-line journey of 828 km through the Strait of Hormuz.

**Fig 7:**
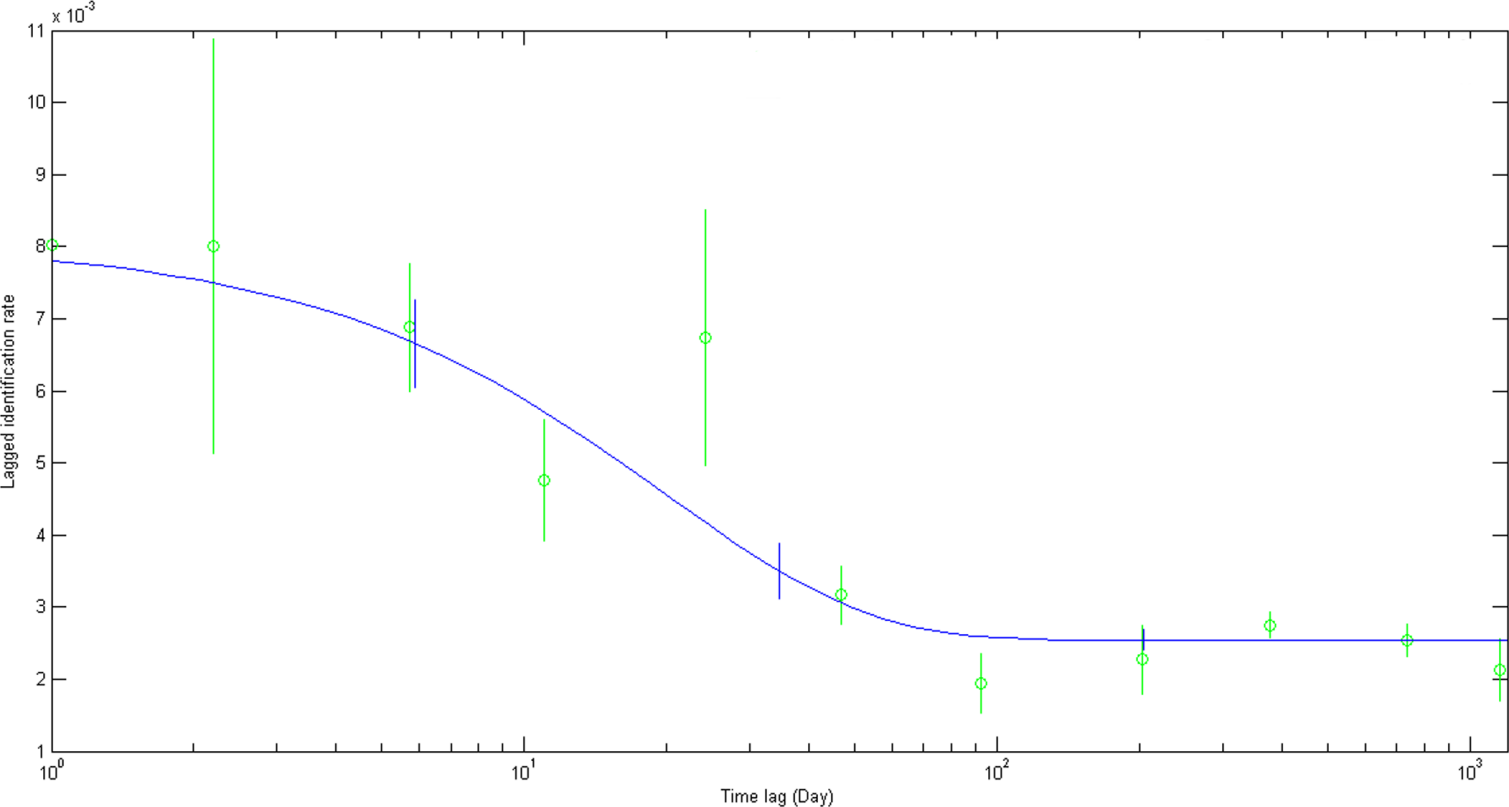
The probability of re-identifying an individual whale shark over time (LIR; mean ± S.D.) within Al Shaheen compared to the best-fitting movement model.

Modelled LIR gradually declined from 1 to 100 days (Fig 7), suggesting that most sharks left the area after a short period of residency, but there was a slight increase after approximately one year, indicating periodic return to the study area by some sharks. Model G was the best fit to the empirical data based on QAIC (Table 2). In this model scenario, there were an estimated 123.72 sharks ± S.E. 15.3062 (95% C.I. 95.3501–152.3211) within Al Shaheen on any given day. The mean residency time within Al Shaheen was 28.78 days ± S.E. 9.3522 (95% C.I. 8.7195–46.5447), and 62.74 days ± S.E. 16.7609 (95% C.I. 22.6607–86.947) outside of Al Shaheen.

**Table 2:** Model parameters and comparison for the Lagged Identification Rate of whale sharks.

Model	Model descriptions for Al Shaheen	ΔQAIC
A	Closed	17050
B	a1 = N	6334
C	Emigration/mortality	6325
D	Closed: emigration+reimmigration	6323
E	a1 = N; a2 = Mean residence	6325
F	Emigration+reimmigration+mortality	6317
G	a1 = N; a2 = res time in; a3 = res time out	6270
H	a1 = N; a2 = res time in; a3 = res time out; a4 = mortality	6298
	**Model descriptions for LIR between sub-areas**	
I	Fully Mixed (a1)	0.0054
J	Fully Mixed (a1 = N)	0
K	Migration—Full Interchange (a1 = diffusion rate from area 1 to area 2; a2 = 1/N)	2.0054
L	a1 = N; a2 = mean residence time in area 1	2

N = Population

Lagged Identification Rate was then redefined to model movement between two distinct sub-areas: (1) Al Shaheen, and (2) the Gulf of Oman including the Strait of Hormuz. The fully mixed model J (a1 = N) was the best fit (Table 2), although both fully-mixed models had a high degree of support. These results indicate that sharks regularly move between the Arabian Gulf and northern Gulf of Oman over inter-annual timescales. The estimated population size for this entire area was 2837 sharks ± 1243.9 S.E. (95% C. I. 1720–6295.0).

Sharks were most likely to be re-sighted again within the same area, but there was also a chance of sighting an individual across all areas (including outside the study region; Table 3).

**Table 3:** The probability of a shark originally identified from an area being re-sighted within a different area.

	To Area			
		Al Shaheen	Gulf of Oman	Outside
	**Al Shaheen**	0.9093	0.0338	0.0569
**From Area:**	**Gulf of Oman**	0.1118	0.8856	0.0025
	**Outside**	0.1364	0.2625	0.6011

## Discussion

The Al Shaheen field off Qatar, where large aggregations of whale sharks occur in the boreal summer months, is the main hotspot for whale shark occurrence within the Arabian Gulf and region. Sharks aggregate here to feed on tuna spawn produced by mackerel tuna, *Euthynnus affinis* (Cantor, 1849) [23]. Over 340 whale sharks have been identified from this site since 2011, indicating that the area is a globally-significant feeding area. The majority of whale sharks encountered in Al Shaheen were mature males, and this is the first global site where a significant proportion of mature male individuals have been reported. The second identified regional hotspot was in the Musandam region of Oman. There were high levels of observed and modelled connectivity between the Arabian Gulf and Gulf of Oman, but no connectivity with other Indian Ocean sites was noted. This high connectivity allowed the application of maximum likelihood population models, providing the first regional population estimate for the species: 2837 sharks ± 1243.9 S.E., albeit with broad 95% confidence intervals of 1720 to 6295 sharks.

### Regional occurrence and abundance

Overall, the majority of sharks were encountered in the warm boreal summer months between April and October. No whale shark feeding activity, from anywhere in the study region, has been documented from outside these summer months. The seasonal nature of the aggregation off Qatar matches the seasonal occurrence of whale shark sightings elsewhere around the region. The Strait of Hormuz, situated north of the Musandam region of Oman, is the sole connection between the Arabian Gulf and the Gulf of Oman. These are productive areas with high coral coverage, fish numbers, and diversity. Within the Musandam region, most encounters were recorded from around Lima Rock, particularly from the south side (Fig 3). Lima Rock is accessible by speedboat for ‘day-tripper’ divers from the town of Dibba, increasing diver numbers compared to less accessible sites further north that are only reachable by two or three-day dive trips. Lima Rock is surrounded by an area of comparatively deeper water, and has strong upwelling currents that may make this area more attractive to whale sharks. The Daymaniyat Islands are a popular weekend diving destination for UAE and Oman residents. Whale sharks were most frequently observed on the dive sites on the outside of the island chain, where feeding has also been observed. Although there have been few records of surface feeding within the Gulf of Oman, studies from elsewhere have shown that sub-surface zooplankton can constitute a significant proportion of whale shark diet [2]. It is possible that feeding activities are underreported. One shark was encountered at Octopus Rock in the Musandam and then re-sighted exactly a year later in the same local area. Sharks encountered in Omani waters were re-sighted one, two, three and four years apart and between different local hotspots showing a level of affinity to the area. The majority of recreational diving off Oman occurs during the local weekend, Fridays and Saturdays. Most encounters reported from the Musandam and Daymaniyats occurred on weekends, suggesting that more sharks would be encountered if diving activity increased over weekdays.

Within UAE waters, the majority of encounters with whale sharks took place at popular coastal dive sites on the East coast. Similar to the Musandam, whale sharks have not been reported to feed here or be resident to the area. However, one feeding aggregation was reported from 35 km offshore. With a single exception, encounters from the west coast of the UAE were from marinas or ports along the coast. Several dive companies frequently dive the waters of the west coast of the UAE, but no whale sharks have been reported to date. One shark was reported from a UAE platform worker from the Salman Oil Field. Similarly, whale sharks were rarely reported from Qatari waters outside of Al Shaheen, with only one observation from mainland Qatar. These sighting data, viewed as a whole, indicate that whale sharks rarely use the shallower coastal waters around the Gulf. As regional whale shark encounters were mainly recorded from popular recreational areas, data reported may be skewed towards these areas and underestimate occurrence along this coastline.

The rapid increase in reports and successful identification of sharks by seafaring individuals from 2004 to 2013 can be attributed to increased diving activity, an increase in the number of underwater cameras in use, and improving awareness of how to report and submit an encounter. The Sharkwatch Arabia project focused on engaging divers and dive centers through social media such as Facebook and Twitter. These sites allow for easy sharing of images and information. As social media presence and online networking tools grow, so will the reach and success of citizen science initiatives such as Sharkwatch Arabia.

### Population ecology in Al Shaheen, Qatar

The majority of regional encounters took place in the Al Shaheen area of Qatar, a major aggregation site for whale sharks [23]. Three hundred and forty-one sharks were identified here between 2011 and 2014, with a modelled estimate of 124 sharks present each day over the aggregation season. Sharks varied in size from 4 to 10 m TL, with laser photogrammetric measurements comparable to visual length estimates. The aggregation is male dominated, with a median length of 8 m. Sharks below 5 m or in excess of 9 m length were rarely encountered. A high proportion of adult sharks, particularly males, was documented. This is unique amongst the predictable feeding aggregations examined to date, which are typically biased towards juvenile males [17,19,24–26,59]. The 7.29 m TL_50_ for male maturity was similar to the 7 m estimate from Quintana Roo in Mexico [36], lower than that reported from Western Australia (8 m, [28]) and Mozambique (9. 16 m, [27]). Although Indian Ocean-level genetic population structure has not been shown in whale shark [60], these differences in maturity suggest that limited broad-scale mixing may occur.

Two 9 m TL female sharks were visually assessed to be pregnant. To date suspected pregnant females have only been reported from Galapagos, the Gulf of California and the Gulf of Mexico [31–33], while one confirmed pregnant female was examined in Taiwan [6]. To our knowledge, Al Shaheen is the only site in the Indian Ocean basin where pregnant female sharks have been observed. Size of maturity has also been estimated to be around 9 m in the Eastern Pacific [32,36]. There have been several reports of neonatal sharks off the Balochistan coast in Pakistan and the discovery of a 58.6cm free swimming neonate suggests that whale sharks do give birth in the Northern Indian Ocean [61].

Modelled re-sighting data estimated a mean residency time of 29 days within Al Shaheen. This was similar to the results of mark-recapture modelling of whale sharks from Ningaloo Reef, where mean residency was estimated to be 33 days [62], and the estimated 24–33 days of sharks off Quintana Roo in Mexico [33]. These latter two sites, which are noted feeding areas for whale sharks [63,64], contrast with the relatively short estimated 12 day residency at Utila, where sharks are feeding opportunistically on baitfish [42]. A high degree of inter-annual site fidelity was also evident amongst Al Shaheen sharks, with 41% of sharks seen in more than one year, even with the limited number of sampling trips that were possible. This re-sighting rate was higher than most other whale shark aggregation sites, e.g. 22% in Utila, Honduras [42], 13% in Holbox, Mexico [29], 23% in Djibouti and 28% in Seychelles [26], and 35% in Western Australia [62].

Surface water temperatures within the Arabian Gulf frequently exceed 35°C [53]. Water temperatures from zero to 10 m depth at Al Shaheen, where the sharks are feeding for extended periods of time, can exceed 33°C during the hottest months [23]. Berumen et al. [22] recorded temperatures ranging from 8°C to 34°C from satellite-tagged sharks in the Red Sea. Whale shark distribution appears to be limited by <21°C surface temperatures [65,66], but their upper tolerance is not yet known. The presence of large numbers of whale sharks surface feeding within the Arabian Gulf during the warm summer months for extended periods of time shows that whale sharks are able to tolerate temperatures in excess of 30°C for hours at a time. There is evidence for behavioural thermoregulation influencing whale shark dive patterns in cooler temperatures [67], and they may seek to cool their internal temperature in warmer waters [68]. Al Shaheen is likely to be the aggregation site where whale shark experience the warmest water temperature, so a more detailed evaluation of their coping strategies may provide insight into their long-term response to a warming ocean climate.

### Regional connectivity and population size

Within the Indian Ocean, limited international connectivity has previously been observed [17,59,62], which has precluded the estimation of population size at a regional scale. In the present study, whale sharks were recorded and re-sighted in all significant regional hot spots, suggesting that individual sharks are moving freely between the Arabian Gulf and Gulf of Oman. The largest number of shared individuals was noted between Musandam and Al Shaheen, the two areas with the greatest number of recorded encounters (Fig 2). Model selection identified a fully-mixed model as the most likely scenario. Regional population size was estimated at 2837 ± 1243.9 S.E. The broad 95% confidence intervals bounding this estimate, 1720 to 6295 sharks, indicate that this should be treated as a rough approximation. However, as the first broad-scale population estimate that has been obtained for whale shark, it is an important step towards understanding relative abundance of this species and developing management strategies for the conservation of this globally threatened species.

## Conclusions

Research in Al Shaheen began comparatively recently, in 2011, and has already documented high abundance, long residency time and philopatric behaviour to the site. The reason for the unique adult male bias observed in the Al Shaheen feeding aggregation has not been identified, but the sex and size-based segregation inherent in whale shark aggregations globally makes this an interesting topic to investigate. The high connectivity, and relatively small regional population size makes the quantification and mitigation of human threats, and potential management measures, a priority within the region.

## Supporting Information

S1 Fig An image of a large female whale shark and researcher taken in Al Shaheen. (TIF)

S2 Fig An image of the remote and mountainous Musandam Governorate of Oman. (TIF)

S3 Fig An aerial image of a whale shark aggregation in the Al Shaheen area showing typical density of feeding sharks and variation in size. (TIF)

S1 Data Whale shark encounter reports in the Arabian Gulf and Gulf of Oman up to and including 2014. (XLS)
