# Supplementary figures and images for: Population Structure, Abundance and Movement of Whale Sharks in the Arabian Gulf and the Gulf of Oman
Source: PLoS One. 2016 Jun 30;11(6):e0158593. doi: 10.1371/journal.pone.0158593 (PMC4928964; doi:10.1371/journal.pone.0158593)

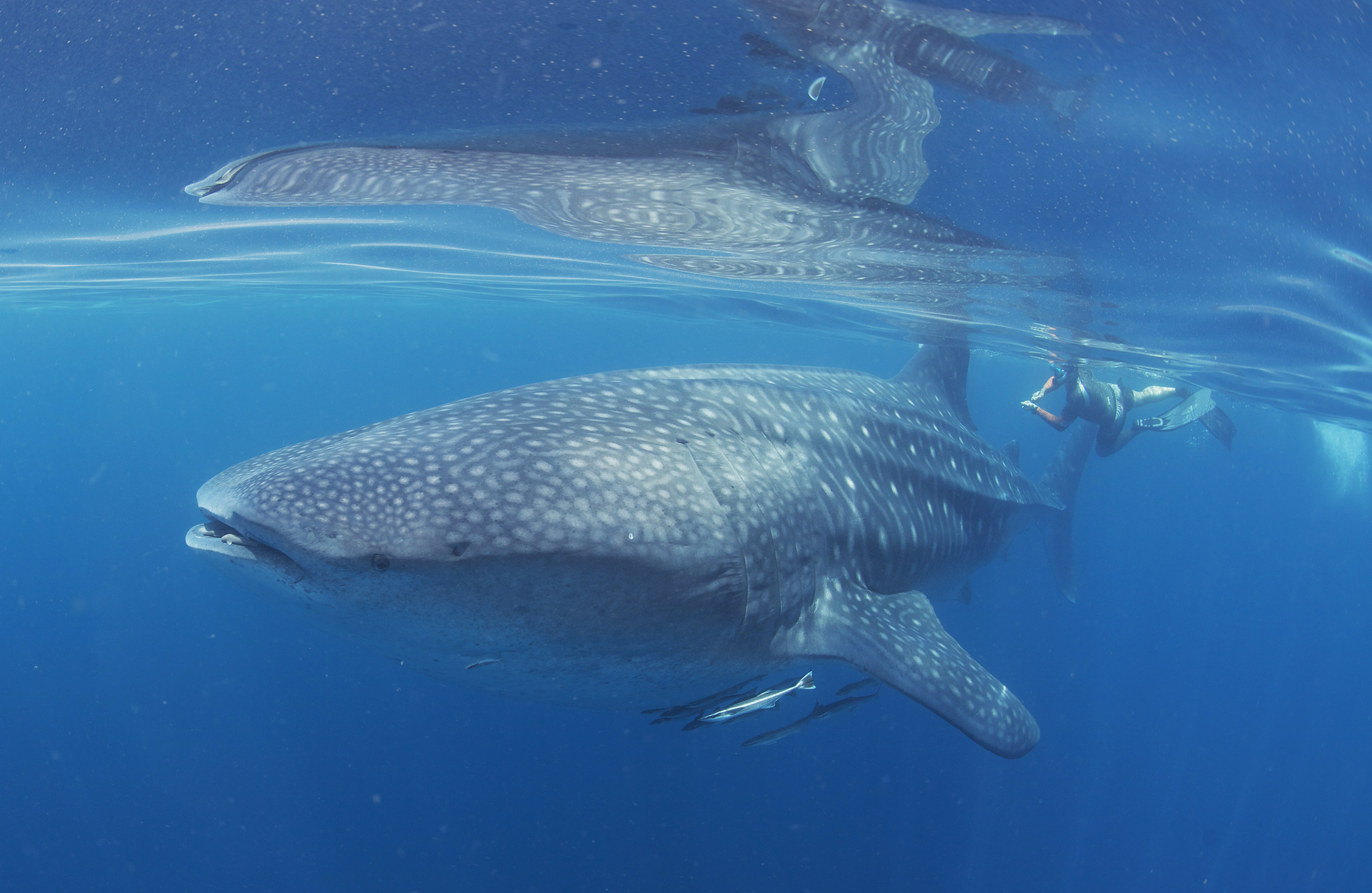

Supplement: S1 Fig — (TIF) [file pone.0158593.s001.tif]

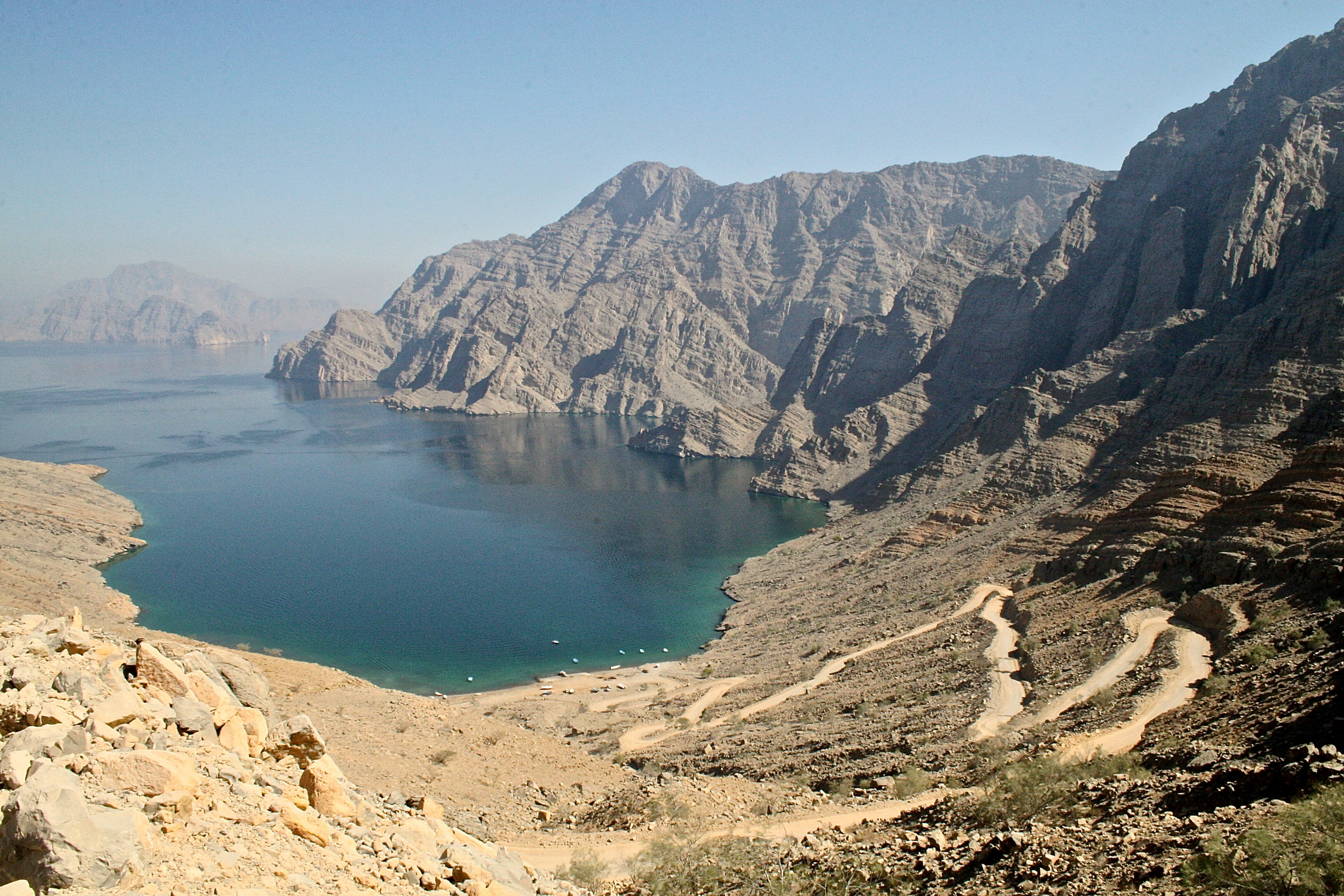

Supplement: S2 Fig — (TIF) [file pone.0158593.s002.tif]

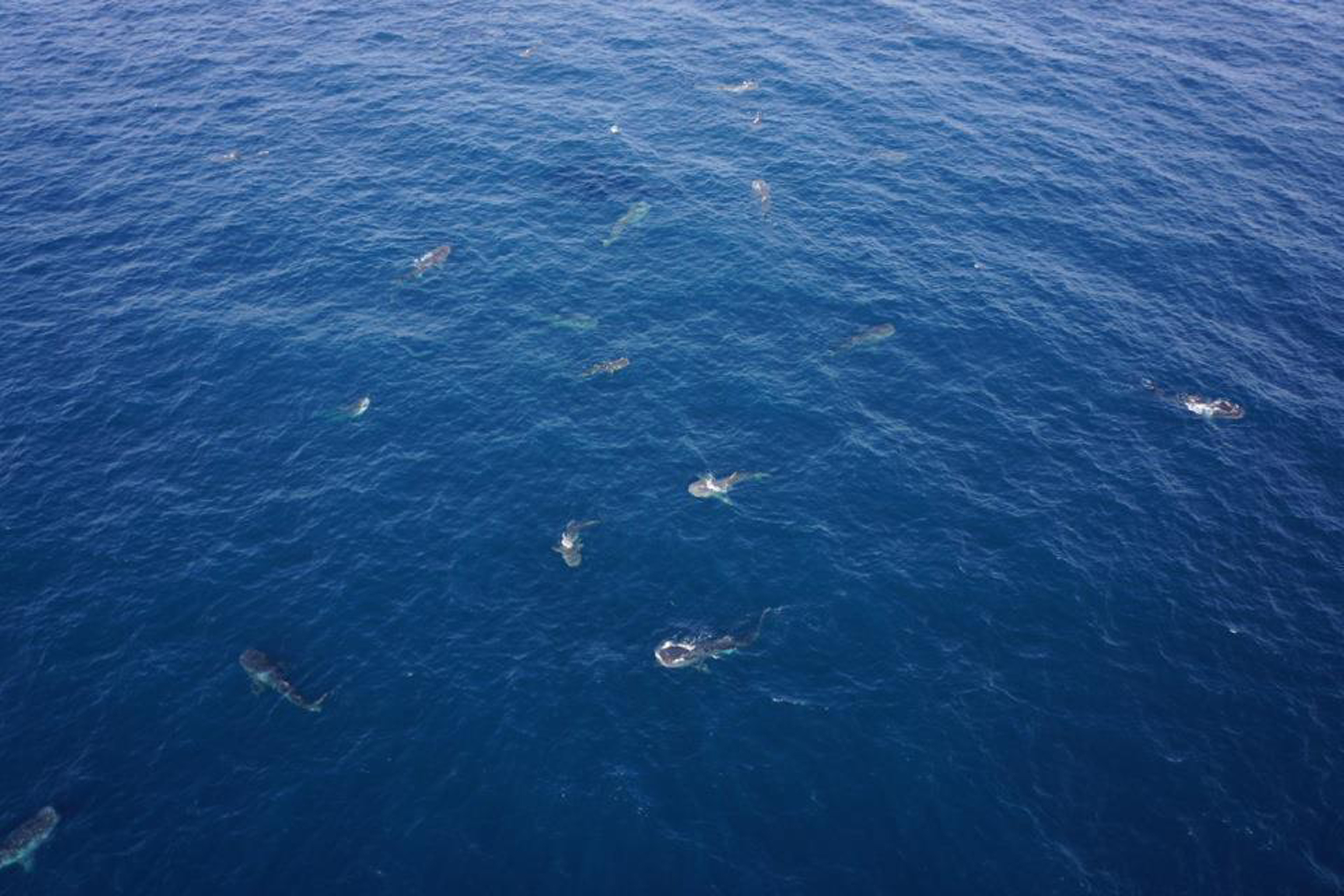

Supplement: S3 Fig — (TIF) [file pone.0158593.s003.tif]
